# Long non-coding RNA LINC01207 promotes cell proliferation and migration but suppresses apoptosis and autophagy in oral squamous cell carcinoma by the microRNA-1301-3p/lactate dehydrogenase isoform A axis

**DOI:** 10.1080/21655979.2021.1972784

**Published:** 2021-10-06

**Authors:** Xiaolin Lu, Liling Chen, Yang Li, Rong Huang, Xiangfeng Meng, Fangfang Sun

**Affiliations:** a Department of Maxillofacial Surgery, Nanjing Stomatological Hospital Medical School of Nanjing University, Nanjing, China; b Department of Prosthodontics, Nanjing Stomatological Hospital Medical School of Nanjing University, Nanjing, China; c Department of Prosthodontics, Pudong New District Hospital of Traditional Chinese Medicine, Shanghai, China

**Keywords:** OSCC, autophagy, linc01207, miR-1301-3p, ldha

## Abstract

Long noncoding RNAs (lncRNAs) have been reported to participate in the progression of various cancers, including oral squamous cell carcinoma (OSCC). This study aims to find out whether lncRNA LINC01207 regulates the progression of OSCC. Reverse transcription quantitative polymerase chain reaction (RT-qPCR) was conducted to evaluate gene expression in OSCC cells and tissues. Cell viability, proliferation, migration, apoptosis, and autophagy were detected using Cell Counting Kit-8 (CCK-8), colony formation, Transwell assays, flow cytometry, and western blot analysis. Luciferase reporter and RNA immunoprecipitation (RIP) assays were conducted to assess the interactions among genes. We found that LINC01207 was overexpressed in OSCC cells and tissues. LINC01207 silencing inhibited OSCC cell proliferation and migration but promoted apoptosis and autophagy, and LINC01207 overexpression had an opposite result. LINC01207 interacted with microRNA-1301-3p (miR-1301-3p) while lactate dehydrogenase isoform A (LHDA) was targeted by miR1301-3p. Effects caused by LINC01207 downregulation on OSCC cells were reversed by overexpression of LDHA. Overall, LINC01207 promotes OSCC progression via the miR-1301-3p/LDHA axis

## Introduction

Oral squamous cell carcinoma (OSCC), ranking the sixth most common cancer worldwide, accounts for more than 90% of head and neck cancer [1,2]. OSCC is prone to distant metastasis, such as lung metastasis, bone metastasis, lymphatic metastasis, and blood metastasis [3]. There are over 300, 000 new cases of OSCC each year worldwide, and more than 140,000 patients die of OSCC each year [4,5]. Although great progress has been made to treat OSCC over the last years, the 5-year survival rate of OSCC patients has remained approximately 50% without any significant progress [6]. Hence, it is urgent to investigate novel clinical biomarkers that potentially function as targets for diagnosis or therapy of OSCC.

Autophagy is defined as the degradation of components via the autophagosome and lysosome [7,8]. The soluble autophagosome light chain 3 (LC3I) is the autophagosome related form (LC3II) during the autophagosome elongation process [9]. LC3II is degraded along with other components of autophagosome after fusion with lysosome. Therefore, the production of LC3II or the transformation of LC3 is regarded as indicator of autophagy activity [10]. Autophagy-related gene 7 (ATG7) composes part of the conjugation system involved in the redistribution of LC3II to the phagophore. The conjugation system consisting of ATG12-ATG5-ATG16 is pivotal for autophagosome elongation [11]. Evidence shows that autophagy is a major event in tumor progression [12].

Long noncoding RNAs (lncRNAs) consist of a subset of noncoding RNA transcripts that are more than 200 nucleotides in length [13–15]. LncRNAs play vital roles in gene regulation, genomic stability, survival, proliferation, and migration of cancer cells [16]. Studies have demonstrated that lncRNAs are closely related to the occurrence and progression of OSCC. For example, LINC00958 promotes OSCC cell proliferation, induces cell death and reduces autophagy [17]. LncRNA SNHG20 regulates OSCC cell migration, invasion, and proliferation via the microRNA-19b-3p/RAB14 axis [18]. Given that OSCC is the most common type of head and neck cancer, we selected several lncRNAs that were reported to exert important effects in head and neck cancer for the purpose of investigating the role of lncRNA in OSCC. We successfully screened out LINC01207 as the most significantly upregulated lncRNA in OSCC tissues. LINC01207, located in the genomic 4q32 locus, is reported to be upregulated in some cancers, and its downregulation could inhibit tumor growth and promote apoptosis [19,20]. The ceRNA (competitive endogenous RNA) mechanism is a common post-transcriptional mechanism in which lncRNAs act as the endogenous sponge of microRNAs (miRNAs) to free the inhibition of miRNAs on message RNAs (mRNAs). It has been widely reported that lncRNAs regulate OSCC progression via the ceRNA pattern [21,22]. Additionally, LINC01207 promotes head and neck squamous cell carcinoma cell proliferation and stemness characteristics by acting as a ceRNA for miR-5047 [23]. LINC01207 contributes to prostate cancer progression by downregulating miR-1972 and upregulating LIM and SH3 protein 1 [24]. Therefore, we inferred that LINC01207 may function as a ceRNA in OSCC.

Lactate dehydrogenase A (LDHA) is a major molecular mediator of the Warburg effect and plays a critical role in the metabolism of tumor cells [25,26]. It has been reported that elevated expression of LDHA is a hallmark of many tumors and is associated with the clinicopathological features and survival outcomes of patients [27,28]. Inhibition of LDHA typically results in accelerated oxygen consumption, reduced cell malignant transformation and markedly delayed tumor formation, indicating the underlying role of LDHA in tumor initiation or maintenance [29].

In the present study, we are aimed at exploring the biological role of LINC01207 in OSCC. Due to its higher expression in tumor tissues than in normal tissues, we hypothesized that LINC01207 may play an oncogenic role in OSCC. Subsequently, a series of molecular experiments were performed to test whether LINC01207 acts as a ceRNA in OSCC. Our study may provide a promising therapeutic target for the treatment of OSCC.

## Materials and methods

### Tissue samples and cell lines

The OSCC samples (n = 30) and adjacent normal tissues (n = 30) were obtained from OSCC patients at Nanjing Stomatological Hospital Medical School of Nanjing University (Jiangsu, China) and then stored at −80°C. No other anticancer treatment was given to patients before the surgery. All patients provided written informed consent. This study was approved by the Ethics Committee of Nanjing Stomatological Hospital Medical School of Nanjing University (Jiangsu, China).

The OSCC cell lines, containing HSC-3 and HSC-4 cells, and human normal oral keratinocytes (NOK) cells were purchased from the Cell Bank of Chinese Academy of Science (Shanghai, China), and were incubated in Dulbecco’s Modified Eagle Medium (Gibco, Thermo Fisher Scientific, Inc., USA) added with 10% fetal bovine serum and 1% streptomycin (Sigma, St Louis, MO, USA). All the cells were maintained in a humidified atmosphere at 37°C containing 5% CO_2_.

### Cell transfection

The short hairpin RNAs (shRNAs) targeting LINC01207 and nonspecific shRNAs (sh-NC), as well as pcDNA3.1-LINC01207/LDHA and empty vector were purchased from GenePharma (Shanghai, China). The miR-1301-3p mimics and negative control (NC) mimics were obtained from Genechem (Shanghai, China). HSC-3 and HSC-4 cells were seeded in 6-well plates at a density of 5 × 10^5^ per well. When cell confluence reached 70–80%, the transfection was performed using Lipofectamine 3000 (Invitrogen, Carlsbad, CA, USA) for 48 h according to the manufacturer’s protocol [30].

### Reverse transcription quantitative polymerase chain reaction (RT-qPCR)

Total RNAs were extracted using TRIzol reagent (Takara, Shiga, Japan) following the instructions of manufacturer. First-strand cDNA was obtained from 2 μg total RNA using PrimeScript RT reagent (Takara). Subsequently, PCR was performed on SteponePlus Real-Time PCR Systems (Applied Biosystems, Foster City, CA, USA) with SYBR Green (Sigma) with glyceraldehyde-3-phosphate dehydrogenase (GAPDH) and U6 as internal references. The relative expression was calculated using the 2^−ΔΔCt^ method [31]. Primers used in RT-qPCR are as follows: GAPDH forward 5ʹ-CATGAGAAGTATGACAACAGCCT-3ʹ and reverse 5ʹ- AGTCCTTCCACGATACAAAG-3ʹ, U6 forward 5ʹ- ATACAGAGAAAGTTAGCACGG-3ʹ and reverse 5ʹ- GGAATGCTTCAAAGAGTTGTG-3ʹ, LINC01207 forward 5ʹ-CTGAAGACTCTGTTTGAATCAG-3ʹ and reverse 5ʹ-AAACTTCTTCACCAGAAGCA-3ʹ, miR-1301-3p forward 5ʹ-TTGCAGCTGCCTGGGAGTGACTTC-3ʹ, LDHA forward 5ʹ-TTCCAGTGTGCCTGTATGG-3ʹ and reverse 5ʹ-TTATCAGTCCCTAAATCTGGGTG-3ʹ.

### Cell Counting Kit-8 (CCK-8) assay

CCK-8 Kit (Beyotime, Shanghai, China) was used to assess cell viability as previously described [32]. After being transfected for 48 h, HSC-3 and HSC-4 cells (5000 cells per well) were seeded in 96-well plates. Next, 10 μl CCK8 reagent was added to each well at 0, 24, 48, and 72 h, respectively. A microplate reader (SpectraMax i3, Molecular Devices, USA) was utilized to detect cell viability by measuring the absorbance at 450 nm.

### Colony formation assay

After the abovementioned transfection, HSC-3 and HSC-4 cells were seeded in 6-well plates (500 cells/well). After 14 days of incubation, the colonies were then fixed with 4% paraformaldehyde for 30 min and then incubated with 0.5% crystal violet (Beyotime, Shanghai, China) for 1 h at room temperature. And finally, the number of colonies with more than 50 cells was counted [33].

### Cell migration assay

Cell migration was examined using Transwell inserts (pore size, 8.0 µm; Corning, USA) as previously described [34]. Briefly, 2 × 10^5^ cells/well cells in 200 µl serum‑free medium were suspended in the upper chamber, and the lower chamber was filled with 700 µl medium containing 15% fetal bovine serum. After 24 h at 37°C in an atmosphere containing 5% CO_2_, the membranes in the lower chamber were fixed in 4% paraformaldehyde at room temperature and stained with crystal violet for 20 min. After washing, images of the cells on the membranes were captured under a light microscope in five randomly selected fields per sample.

### Flow cytometry

Annexin V-fluoresceine isothiocyanate (FITC)/Prodium Iodide (PI) double-labeled staining kit (BD Biosciences, San Jose, CA, USA) was used in this assay. The procedure was performed as previously described [35]. The cells (2 × 10^5^/well) in 6-well plates were collected, washed twice with cold phosphate buffered saline, and resuspended in 1 × binding buffer. Subsequently, cells were stained with 10 μl Annexin V-FITC for 15 min and 5 µl PI for 10 min in the dark at room temperature. Cells were examined using the FACSCanto II flow cytometer (BD Biosciences). Analysis of flow cytometry data was performed using FlowJo version X.10.0.7–1 (FlowJo, LLC).

### Western blot

Western blot was performed using standard and established protocol as previously published [36]. Proteins were extracted from OSCC cells using radioimmunoprecipitation assay lysis buffer (Beyotime, Shanghai, China). Protein concentration was quantified using a bicinchoninic acid assay kit (Beyotime).

In this assay, an equal amount of protein extraction was separated by 10% sodium dodecyl sulfate polyacrylamide gel and then transferred into polyvinylidene fluoride membranes (Millipore, USA). Afterward, the membranes were blocked in 5% nonfat milk and cultured at room temperature for 1 h. The membranes were incubated overnight at 4°C with the following primary antibodies: anti-ATG5 antibodies (ab108327, Abcam, Cambridge, UK), anti-p62 antibodies (ab109012, Abcam), anti-LC3-I antibody (ab192890, Abcam), anti-LC3-II antibodies (ab192890, Abcam), anti-GAPDH (ab181602, Abcam), and anti-LDHA antibodies (ab101562, Abcam). Afterward, the membranes were incubated with secondary antibody goat anti-rabbit IgG (ab205718; Abcam). Finally, the enhanced chemiluminescence kit (GE Healthcare Bio-Sciences, Pittersburg, PA, USA) was utilized to visualize the proteins. Protein bands were detected by the Image J software (National Institutes of Health, Bethesda, MA) based on the intensity.

### Fluorescence In Situ Hybridization (FISH)

The LINC01207 FISH probe was designed and synthesized by RiboBio (Guangzhou, China). Briefly, cells were seeded on coverslips, fixed with 4% paraformaldehyde for 15 min at 4°C, and permeabilized with 1% Triton X-100 for 30 min. Next, the coverslips were washed with 2 × SSC (300 mM NaCl, 30 mM sodium citrate, pH 7.0) for 30 min at 37°C and dehydrated with ethanol at room temperature. FISH probes in hybridization buffer were incubated with coverslips overnight at 37°C. Subsequently, coverslips were washed twice with 0.4 × SSC/0.3% Tween20 and 2 × SSC/0.1% Tween20, stained with 4ʹ,6-Diamidino-2ʹ-phenylindole, and then detected by a confocal fluorescence microscope (Leica, Germany) [37].

### Luciferase reporter assay

The wild type (Wt) or mutant (Mut) sequence in the LDHA 3ʹUTR containing the predicted binding site for miR-1301-3p was synthesized to generate the fragment of LDHA-wild type (LDHA-Wt) and fragment of LDHA-mutant (LDHA-Mut). The targeted fragments were inserted into the pmirGLO vector (Promega, Madison, WI, USA). Then the recombinant plasmids (LDHA-Wt and LDHA-Mut) were transfected with miR-1301-3p mimics or NC mimics into cells, respectively. Similarly, miR-1301-3p-Wt/Mut plasmids were transfected with pcDNA3.1 or pcDNA3.1-LINC01207 into OSCC cells. After 48 h, the luciferase activity was examined using the Dual-luciferase Reporter Assay System (Promega) [38].

### RNA immunoprecipitation (RIP) assay

RIP was performed using a Magna RNA-binding protein immunoprecipitation kit (EMD Millipore, Billerica, MA, USA) [39]. At 90% confluence, cells were centrifuged at 4°C for 5 min at 1,000 × g, washed with pre-cooled phosphate buffered saline and lysed with radioimmunoprecipitation assay lysis buffer. Subsequently, the lysates were incubated for 10 min at 4°C with human Ago2 antibody (ab186733; Abcam; 5 µg) conjugated on magnetic beads, with IgG antibody (ab172730; Abcam; 5 µg) as the control group. Samples were treated with Proteinase K for 30 min at 55°C with gentle agitation. Immunoprecipitated RNA was isolated using TRIzol. Co-precipitated RNAs were purified, identified, and analyzed with RT-qPCR.

### Statistical analysis

Data were analyzed using SPSS 19.0 software (SPSS Inco, Chicago, IL, USA) and are presented as the mean ± standard deviation. One-way or two-way analysis of variance and student’s t-test were utilized to compare the differences between groups. Linear correlation analysis was performed using Spearman’s correlation coefficient. Data were statistically significance when p < 0.05.

## Results

In this study, we investigated the biological role and studied the molecular mechanisms of LINC01207 in OSCC. Our data showed a significant upregulation of LINC01207 in OSCC tissues and cell lines. LINC01207 overexpression promoted the malignant phenotypes of OSCC cells, and inhibited cell apoptosis and autophagy. LINC01207 upregulated LDHA expression by acting as a ceRNA for miR1301-3p. Overall, our study demonstrated a functional role of the LINC01207/miR-1301-3p/LDHA axis in OSCC, implying that targeting these molecules could be a novel approach in OSCC treatment.

### LINC01207 is overexpressed in OSCC tissues and cells

**Figure 1.**
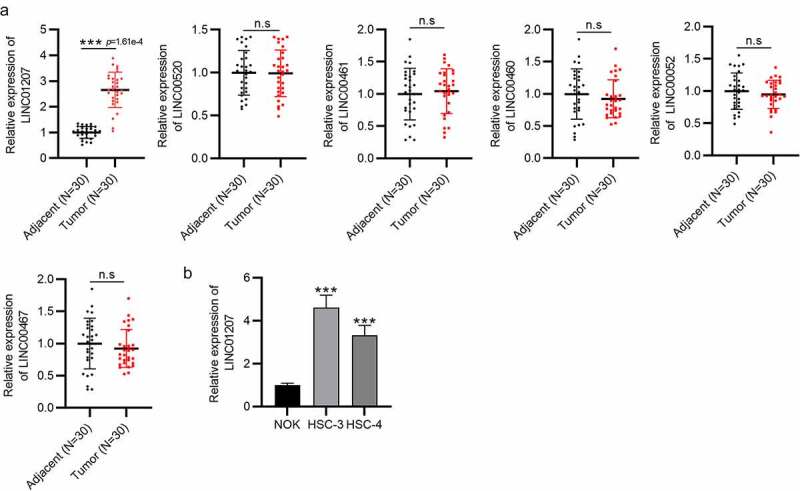
LINC01207 is overexpressed in OSCC tissues and cells. (a) RT-qPCR was conducted to detect the expression of LINC00520, LINC00461, LINC00460, LINC00052, LINC00467, and LINC01207 in OSCC tissues and adjacent normal tissues. (b) LINC01207 expression in NOK, HSC-3, and HSC-4 cells was measured using RT-qPCR. ***P < 0.001

Given that OSCC is the most common type of head and neck cancer, we selected several lncRNAs (LINC00520, LINC00461, LINC00460, LINC00052, LINC00467, and LINC01207) that were reported to exert important effects in head and neck cancer to investigate the role of lncRNA in OSCC. RT-qPCR presented that LINC01207 expression was significantly upregulated in OSCC tumor tissues compared to that in adjacent normal tissues, while the other lncRNAs showed no significant changes (Figure 1(a)). Similarly, the higher level of LINC01207 in HSC-3 and HSC-4 cells than in NOK cells was also detected by RT-qPCR (Figure 1(b)). Therefore, we chose LINC01207 for the study.

**Figure 2.**
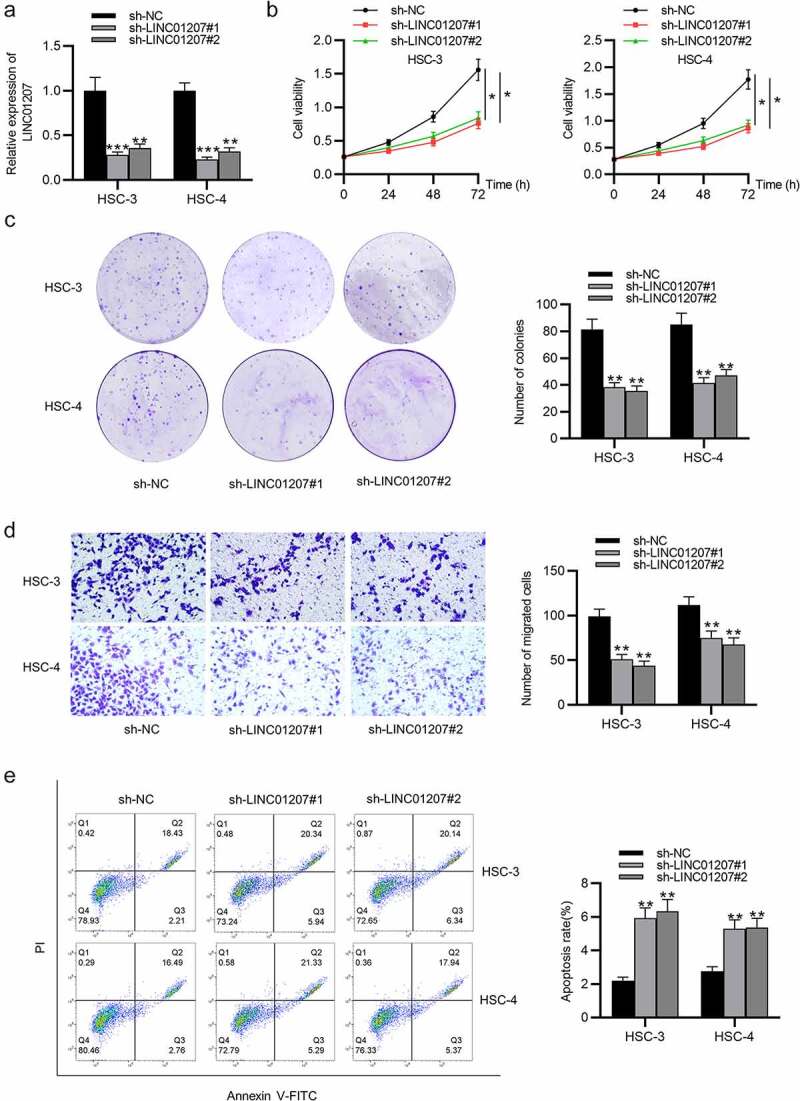
Effects of LINC01207 silencing on OSCC cells. (a) RT-qPCR was used to detect the LINC01207 level in HSC-3 and HSC-4 cells after transfecting sh-LINC01207#1/2. (b) CCK-8 assay was employed to assess cell viability in the transfected cells. (c) the number of colonies after transfection was detected using colony formation assay. (d) transwell assay was used to examine the impact of LINC01207 knockdown in OSCC cell migration. (e) cell apoptosis after transfection was evaluated by flow cytometry. (f) western blot was conducted for the detection of the expression of autophagy-related protein (ATG5, P62, LC3-I/II). *P < 0.05, **P < 0.01, ***P < 0.001

**Figure 2.**
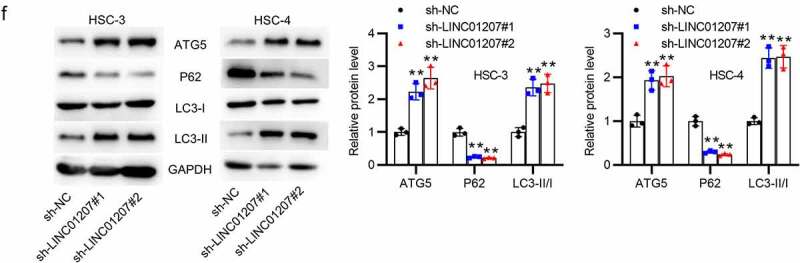
Continued

### LINC01207 silencing inhibits OSCC cell growth and promotes apoptosis and autophagy

**Figure 3.**
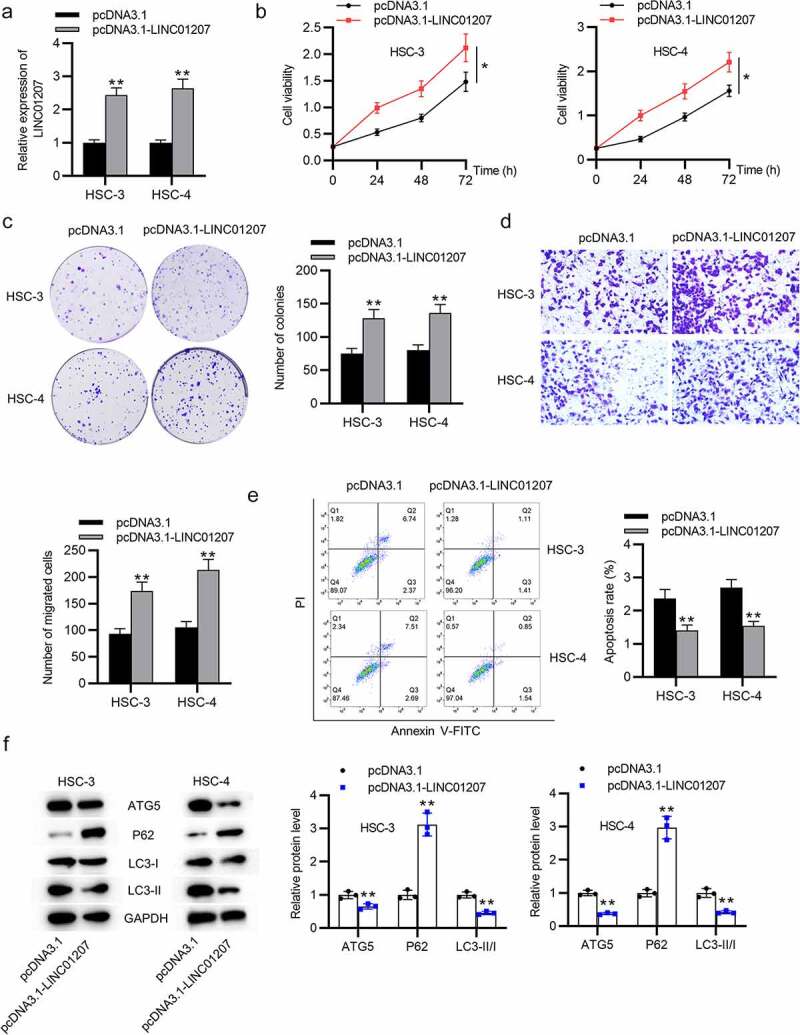
Effects of LINC01207 overexpression on OSCC cells. (a) RT-qPCR was used to detect the LINC01207 level in HSC-3 and HSC-4 cells after transfecting pcDNA3.1-LINC01207. (b) cell viability was detected by CCK-8 assay. (c) the number of colonies was detected using colony formation assay. (d) transwell assay was used to examine the impact of LINC01207 overexpression in cell migration. (e) cell apoptosis was evaluated by flow cytometry. (f) western blot was conducted to measure the expression of autophagy-related protein (ATG5, P62, LC3-I/II). *P < 0.05, **P < 0.01

To verify the function of LINC01207 in OSCC, the following assays were performed. After transfecting sh-NC or sh-LINC01207#1/2 into OSCC cells, as presented by RT-qPCR, LINC01207 expression was significantly downregulated in OSCC cells (Figure 2(a)). The CCK-8 assay demonstrated that after LINC01207 knockdown, the viability in HSC-3 and HSC-4 was inhibited (Figure 2(b)). Consistently, the number of colonies was decreased after transfection of sh-LINC01207#1/2 (Figure 2(c)). Transwell assay was used to examine the impact of LINC01207 in OSCC cell migration, and the results showed that the migration of HSC-3 and HSC-4 cells was significantly suppressed by LINC01207 knockdown (Figure 2(d)). Flow cytometry analysis showed that the apoptotic rate was increased in cells transfected with sh-LINC01207#1/2, which indicated that LINC01207 knockdown promotes OSCC cell apoptosis. (Figure 2(e)). We further performed western blot to test the change of autophagy. As shown, the protein expression of ATG5 and LC3-II was upregulated by LINC01207 silencing while that of P62 was downregulated, suggesting LINC01207 silencing promotes cell autophagy in OSCC (figure 2(f)). We then assessed LINC01207 expression after transfection of pcDNA3.1-LINC01207 and found that LINC01207 was upregulated by pcDNA3.1-LINC01207 (Figure 3(a)). Experiments demonstrated that overexpression of LINC01207 significantly facilitated OSCC cell viability, proliferation, migration, and inhibited apoptosis and autophagy (Figure 3(b-f)). Therefore, LINC01207 acts as an oncogenic gene in OSCC.

### LINC01207 sponges miR-1301-3p

**Figure 4.**
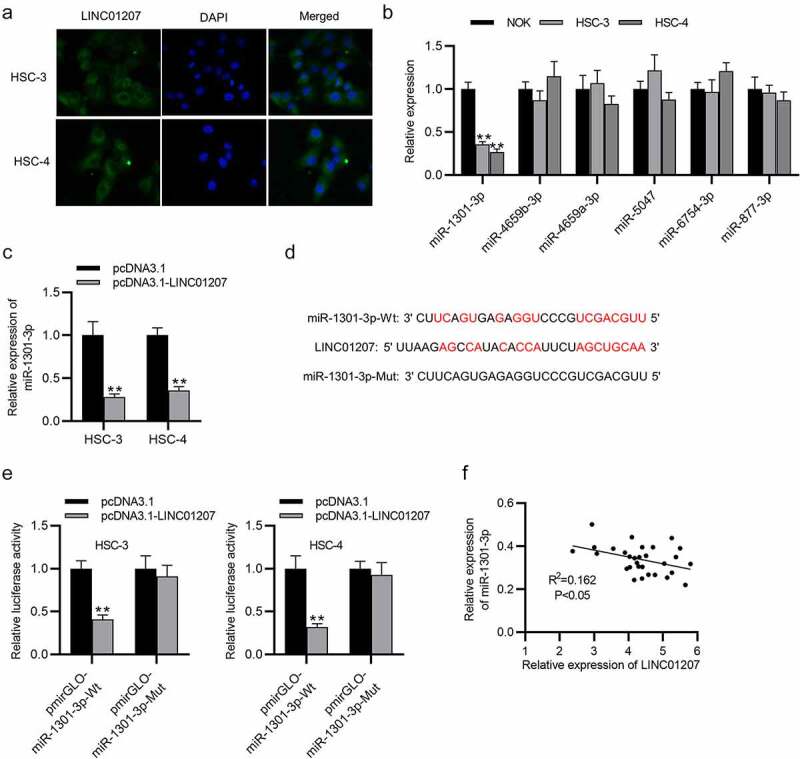
LINC01207 sponges miR-1301-3p. (a) FISH assay was performed to reveal the subcellular location of LINC01207. (b) RT-qPCR was performed to detect the miRNA level in HSC-3, HSC-4, and NOK cells. (c) the miR-1301-3p level in OSCC cells after transfecting pcDNA3.1-LINC01207 was measured using RT-qPCR. (d) the binding site between LINC01207 and miR-1301-3p. (e) after pcDNA3.1 transfection, the luciferase activity of pmirGLO-miR-1301-3p-Wt and pmirGLO-miR-1301-3p-Mut in OSCC cells was detected by a luciferase reporter assay. (f) correlation analysis of the expression of LINC01207 and miR-1301-3p in OSCC tissues. **P < 0.01

Since the subcellular location determines how lncRNA regulates gene expression, we then detected subcellular location of LINC01207 by FISH assay. It was revealed that LINC01207 was significantly distributed in the cytoplasm of HSC-3 and HSC-4 cells (Figure 4(a)). LncRNAs in the cytoplasm are widely reported to function as ceRNAs via sponging miRNAs. The downstream miRNAs of LINC01207 were searched in DIANA (http://carolina.imis.athena-innovation.gr/diana_tools/web/index.php?r=lncbasev2/index-predicted). RT-qPCR presented the expression of miRNAs (miR-1301-3p, miR-4659b-3p, miR-4659a-3p, miR-5047, miR-6754-3p and miR-877-3p) that have predicted binding site for LINC01207, and the results showed that only miR-1301-3p was significantly downregulated in OSCC cells (Figure 4(b)). Additionally, the miR-1301-3p level was reduced after LINC01207 overexpression (Figure 4(c)). The binding site between LINC01207 and miR-1301-3p is presented (Figure 4(d)). Luciferase reporter assay demonstrated that the luciferase activity of pmirGLO-miR-1301-3p-Wt reporters was attenuated after overexpression of LINC01207; however, pmirGLO-miR-1301-3p-Mut reporters had no alternation in the luciferase activity, which suggested that LINC01207 can bind to miR-1301-3p. (Figure 4(e)). Furthermore, correlation analysis showed that there was a negative correlation between LINC01207 and miR-1301-3p expression in OSCC tissues (figure 4(f)). In summary, LINC01207 acts as a sponge of miR-1301-3p.

### LDHA is targeted by miR-1301-3p

**Figure 5.**
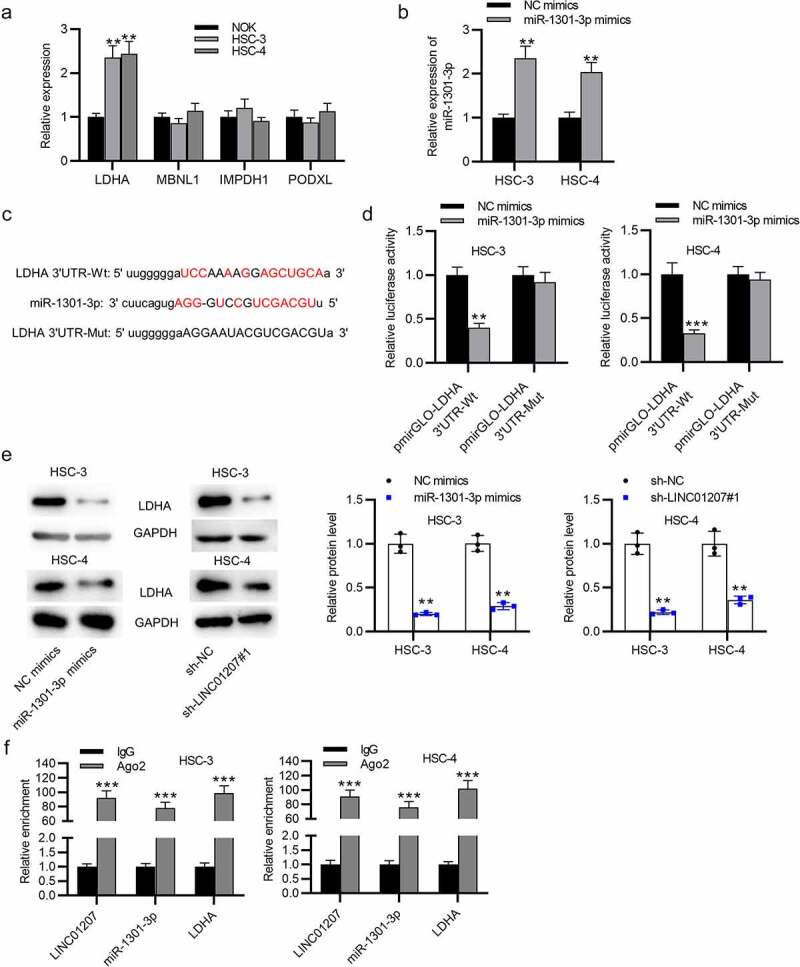
LDHA is targeted by miR-1301-3p. (a) the mRNA expression in OSCC cells was revealed by RT-qPCR. (b) the expression of miR-1301-3p in OSCC cells after transfection with NC mimics and miR-1301-3p mimics was assessed by RT-qPCR. (c) the binding site between miR-1301-3p and LDHA 3ʹUTR. (d) A luciferase reporter assay was conducted to evaluate the luciferase activity of pmirGLO-LDHA 3ʹUTR-Wt and pmirGLO-LDHA 3ʹUTR-Mut in OSCC cells after overexpression of miR-1301-3p. (e) the LDHA protein expression in OSCC cells after miR-1301-3p overexpression or LINC01207 downregulation was measured by western blot. (f) Ago-2 RIP assay was conducted to detect the enrichment of LINC01207, miR-1301-3p and LDHA in RNA-induced silencing complexes. **P < 0.01, ***P < 0.001

The potential targets of miR-1301-3p were predicted in starBase (http://starbase.sysu.edu.cn/) (search category: CLIP Data: strict stringency ≥5; Degradome Data: high stringency ≥3; Predicted Program: PicTar). RT-qPCR presented that LDHA expressed a significantly high level in OSCC cells (Figure 5(a)). Next, miR-1031-3p expression was elevated by using miR-1301-3p mimics (Figure 5(b)). The binding site between LDHA and miR-1301-3p is presented (Figure 5(c)). In HSC-3 and HSC-4 cells, we found that the luciferase activity of pmirGLO-LDHA 3ʹUTR-Wt was reduced when miR-1301-3p was overexpressed while no change was found in pmirGLO-LDHA 3ʹUTR-Mut, showing that LDHA is targeted by miR-1301-3p (Figure 5(d)). Western blot analysis revealed that both downregulation of LINC01207 and upregulation of miR-1301-3p decreased the LDHA protein level in HSC-3 and HSC-4 cells (Figure 5(e)). Furthermore, RIP assay demonstrated that LINC01207, miR-1301-3p, and LDHA were abundantly enriched in the Ago2 group compared to the IgG group (figure 5(f)). Overall, LINC01207 upregulates LDHA through controlling the availability of miR-1301-3p.

### LDHA overexpression reverses the effects of LINC01207 knockdown in OSCC

**Figure 6.**
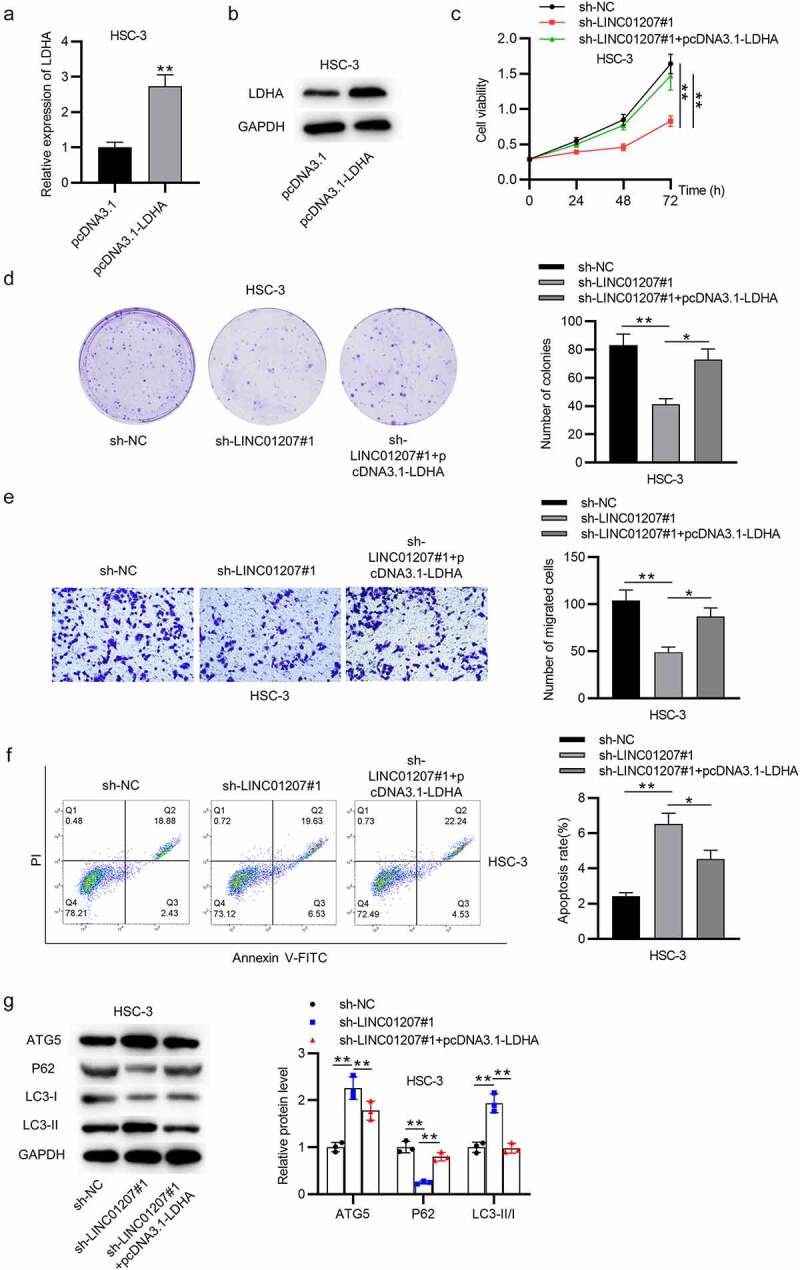
LDHA overexpression reverses the effects of LINC01207 knockdown in OSCC. (a and b) RT-qPCR and western blot analysis of the expression of LDHA in HSC-3 cells after transfecting pcDNA3.1-LDHA. (c-g) after transfecting sh-LINC01207#1 or co-transfecting sh-LINC01207#1 with pcDNA3.1-LDHA in HSC-3 cells, the cell viability was assessed by CCK-8 assay, proliferation was evaluated by colony formation assay, migration was examined by transwell assay, apoptosis was detected by flow cytometry assay, and the protein levels of autophagy-related proteins were presented by western blot. *P < 0.05, **P < 0.01

Since the ceRNA mechanism among LINC01207, miR-1301-3p and LDHA was verified, the rescue assays were in need. RT-qPCR and western blot analysis revealed that LDHA was strongly overexpressed by pcDNA3.1-LDHA in HSC-3 cells (Figure 6(a-b)). CCK-8 and colony formation assays showed that the cell viability and proliferation suppressed by LINC01207 silencing were rescued by LDHA overexpression (Figure 6(c-d)). LINC01207 depletion-mediated suppressive effects on HSC-3 cell migration were reduced after LDHA was overexpressed (Figure 6(e)). Flow cytometry analysis presented that LINC01207 silencing promoted cell apoptosis while LDHA overexpression reversed the results (figure 6(f)). Moreover, the protein expression of ATG5 and LC3-II was upregulated by LINC01207 silencing while was downregulated after LDHA overexpression, and the that of P62 showed an opposite result (Figure 6(g)). Overall, LINC01207 promotes OSCC cellular process by elevating LDHA expression.

## Discussion

In recent years, emerging evidence has revealed that lncRNAs are involved in OSCC progression [40,41]. Moreover, LINC01207 was shown to be an oncogenic gene in tumor progression. LINC01207 downregulation led to the promotion of cell apoptosis and the inhibition of proliferation in gastric cancer [20]. LINC01207 inhibits prostate cancer cell apoptosis and promotes proliferation [24]. In our study, we discovered the overexpression of LINC01207 in OSCC tissues and cells, implying its possible involvement in OSCC progression. We knocked down the expression of LINC01207 in OSCC cells to examine its biological role. Our results revealed that LINC01207 silencing inhibited OSCC cell proliferation and migration but promoted cell apoptosis, and LINC01207 overexpression showed an opposite result. Additionally, a previous study demonstrated that LINC01207 silencing promotes autophagy in pancreatic cancer by increasing LC3II and beclin-1 protein expression while decreasing P62 expression [42]. Similarly, in our study, the inhibitory function of LINC01207 on OSCC cell autophagy was discovered. Therefore, these results suggested that LINC01207 promotes the progression of OSCC in vitro. Animal studies in the future are needed to further confirm whether LINC01207 exerts oncogenic effects in OSCC.

Accumulating evidence suggests that lncRNAs may act as ceRNAs for particular miRNAs to modulate the target genes of the miRNAs, including GC [43,44]. In this study, we assessed the subcellular localization of LINC01207 and observed that majority of LINC01207 were located in the cytoplasm of OSCC cells, which suggested that LINC01207 may function as a ceRNA for sponging miRNA. Thus, we predicted a miRNA that can interact with LINC01207 in OSCC cells and found that LINC01207 has a binding site for miR-1301-3p. MiR-1301-3p has been reported to be downregulated in many tumors and function as a tumor suppressor. Xu G *et al*. found in their study that miR-1301-3p upregulation significantly suppresses colorectal cancer cell growth [45]. The inhibitory effects of miR-1301-3p on breast cancer cell proliferation were found [46]. MiR-1301-3p is downregulated in papillary thyroid cancer and inhibits tumor growth [47]. In this study, we also found that miR-1301-3p was significantly downregulated in OSCC cells, and its expression was negatively correlated to LINC01207 expression. Thus, LINC01207 may play a role in OSCC by sponging miR-1301-3p.

Generally, as a ceRNA, the function of lncRNAs depends on the miRNA targets. LDHA was selected as a direct target of miR-1301-3p through bioinformatics analysis and luciferase reporter assay. LDHA has a key role in tumor cell metabolism and adaptation to unfavorable environmental or cellular conditions [48]. LDHA is overexpressed in prostate cancer and induces a favorable microenvironment for tumor progression [49]. With the deepening of research, our study revealed that LDHA was overexpressed in OSCC cells. Additionally, rescue experiments revealed that LDHA overexpression could rescue the effects caused by knockdown of LINC01207 in OSCC. Moreover, it was reported that LDHA promotes tumorigenesis by facilitating glycolysis and epithelial-mesenchymal transition in OSCC [50], implying the oncogenic property of LDHA in OSCC. Thus, these results demonstrated that LINC01207 acts as a sponge for miR-1301-3p and upregulates the expression of its endogenous target LDHA in OSCC. However, our study has some limitations. The interactions between cytokines in cancer cells are complex, and whether LINC01207 regulates other potential mechanisms will be examined. Additionally, the sample size will be increased to verify the associations between LINC01207 and the prognosis of patients with OSCC.

## Conclusion

Overall, we demonstrated that LINC01207 is upregulated in OSCC tissues and cells. LINC01207 upregulates LDHA expression to promote OSCC cell proliferation, migration, and inhibit apoptosis and autophagy by acting as a ceRNA that sponges miR-1301-3p. Our results provide inspiration for further understanding of the mechanisms of OSCC. In the future work, we will explore the mechanisms of LINC01207 upregulation in OSCC, the correlations between LINC01207 and other miRNAs or proteins, which will further deepen our understanding of the pathogenesis of LINC01207 and make LINC01207 a potential novel diagnostic and therapeutic target for OSCC.
